# Inhibition of p38 mitogen-activated protein kinase phosphorylation decrease tert-butyl hydroperoxide-induced apoptosis in human trabecular meshwork cells

**Published:** 2012-07-26

**Authors:** Yuxia Yang, Xing Liu, Jingjing Huang, Yimin Zhong, Zhen Mao, Hui Xiao, Mei Li, Yehong Zhuo

**Affiliations:** 1 State Key Laboratory of Ophthalmology, Department of Glaucoma, Zhongshan Ophthalmic Center, Sun Yat-sen University, Guangzhou, China; 2 Department of Ophthalmology, the Second People’s Hospital of Foshan, Foshan, China

## Abstract

**Purpose:**

Oxidative stress induced trabecular meshwork cells death is believed to be involved in the pathogenesis and progression of primary open-angle glaucoma (POAG). However, the intrinsic mechanism is yet to be clarified. This study is to investigate the role of p38 mitogen-activated protein kinase (p38MAPK) in *tert*-butyl hydroperoxide (*t*BHP)-induced apoptosis of human trabecular meshwork (iHTM) cells.

**Methods:**

The human trabecular meshwork cells were treated with *t*BHP for 1 or 2 h with or without pretreatment of SB203580, an inhibitor of MAP kinase homologs. Cell viability was analyzed using 3-(4, 5-dimethyl-2-thiazolyl)-2, 5-diphenyl-2h-tetrazolium bromide assay. Reactive oxygen species (ROS) levels were determined using dihydrodichlorofluorescein staining, and the chymotrypsin-like protease activities were measured using the Suc-LLVY-aminoluciferin substrate. Cell apoptosis was analyzed by Hoechst 33258 staining and annexin V-PI labeling. The protein level of phospho-p38 was measured using western blot analysis.

**Results:**

The intracellular ROS increased more than 50 fold and more than 100 fold after *t*BHP exposure for 1 h and 2 h, respectively (p<0.05). However, there was no difference in ROS levels between SB203580(−) and SB203580(+) cells (p>0.05). In 1 h *t*BHP treatment group, the cell viability was significantly improved in SB203580(+) cells (81.08%±1.93%) compared to the SB203580(-) cells (69.35%±1.52%), the chymotrypsin-like proteasome inactivation decreased in SB203580(+) cells (60.94%±0.55%) compared to the SB203580(-) cells (70.59%±0.88%), and apoptosis was impoved in SB203580(+) cells (12.75%±1.91%) compared to the SB203580(-) (28.23%±3.23%) (p<0.05). In 2 h *t*BHP treatment group, cell viability improved in SB203580(+) cells (76.72%±2.11%) compared to SB203580(-) cells (57.88%±2.20%), chymotrypsin-like proteasome inactivation was improved in SB203580(+) cells (62.99%±0.41%) compared to SB203580(-) cells (74.93%±0.54%), and apoptosis was improved in SB203580(+) cells (20.40%±3.44%) compared to SB203580(-) cells (39.20%±5.91%) (p<0.05). Phosphorylation of p38MAPK was significantly increased after tBHP exposure in SB203580 (−) cells and decreased sharply in SB203580(+) cells than that of control group (p<0.05). While there was no difference on the original form of p38MAPK among SB203580(−) and SB203580(+) cells after tBHP exposure and control group (p>0.05).

**Conclusions:**

Activation of p38MAPK plays an important role in *t*BHP-induced apoptosis of iHTM cells. Further study on the mechanisms of p38MAPK in human TM cell apoptosis may help to illuminate the pathogenesis of POAG.

## Introduction

Malfunction of the trabecular meshwork (TM)–Schlemm’s canal (SC) conventional outflow tissue is considered to be one of the main causes of intraocular pressure (IOP) elevation [1-3]. It has been observed that the TM of the patients with primary open-angle glaucoma (POAG) is characterized by morphological and biochemical changes such as loss of TM cells, changes in the cytoskeleton [1], an increase in the extracellular matrix [1,3], and acceleration of senescence [3], which might lead to increased outflow resistance and thus elevated IOP. However, the reasons for these changes are not very clear. Oxidative stress is believed to play an important role in the pathogenesis of POAG [4-6]. It induces characteristic glaucomatous TM changes in vitro, and could be minimized by antioxidants and IOP-lowering substances [7-9]. However, the underlying mechanism of the oxidative stress on TM is as yet unclear.

Mitogen-activated protein kinases (MAPKs) comprise a large family of proteins activated by a wide range of proinflammatory cytokines and environmental stress. MAPKs play pivotal roles in cellular processes such as proliferation, apoptosis, gene regulation, differentiation, and motility [10,11]. MAPKs have four subfamilies: extracellular signal-regulated kinases (ERKs) 1 and 2, ERK5, c-Jun N-terminal kinases (JNKs), and p38 MAPKs, which are proline-directed serine/threonine kinases, and require tyrosine and threonine phosphorylation for activation. Recent studies have shed light on the role of p38MAPK in oxidative stress [12,13]. For example, Kim et al. [14] implied that the phosphorylation of p38MAPK was paralleled by reactive oxygen species (ROS) induction, and this kinase is a critical component of the oxidant stress-sensitive signaling pathways in vascular smooth-muscle cells [15]. Some studies reported that p38MAPK signaling pathway proteins may be involved in the regulation of matrix metalloproteinase-3 [16], or play a role in mechanical stress to TM cells, TM cell senescence [17]. Blockage of the p38MAPK pathway inhibits inducible nitric-oxide (NO) synthase expression in mouse astrocytes [18]; However, no study has examined the role p38MAPK plays in oxidative stress–induced apoptosis in human TM cells.

SB203580, one of the cytokine-suppressive anti-inflammatory drugs, is often used as a p38MAPK inhibitor. Substantial evidence indicates that blockage of p38MAPK with SB203580 can prevent damage caused by oxidative stress [13]. *Tert*-butyl hydroperoxide (*t*BHP) is a common lipid hydroperoxide that causes oxidative stress to cells in vitro [19]. Compared with hydrogen peroxide (H_2_O_2_), *t*BHP is not degraded by catalase; thus, its oxidative effect could be maintained for a longer period of incubation.

Here, we explored whether the p38MAPK pathway can be activated in *t*BHP-induced oxidative stress and apoptosis in immortalized human TM (iHTM) cells. Furthermore, we investigated whether an inhibitor of the p38MAPK pathway (SB203580) could protect against *t*BHP-induced oxidative stress and apoptosis.

## Methods

### Cell culture

The cell line of immortal human trabecular meshwork cells (iHTM) was kindly provided by Dr. Vincent Raymond (Laboratory of Ocular Genetics and Genomics, Quebec, Canada). The cells were cultured in Dulbecco’s modified Eagle’s medium (DMEM; Gibco, Gaithersburg, MD) supplemented with 10% heat-inactivated fetal bovine serum (FBS; Gibco), 2 mM l-glutamine, 100 U/ml penicillin, and 100 μg/ml streptomycin in a humidified 5% CO_2_ incubator at 37 °C. When cells reached 80% confluence, they were detached with 0.25% trypsin solution (Gibco) and collected for the subsequent experiments.

### tBHP and SB203580 treatment

All cells were divided into control and treatment groups. The latter were divided into *t*BHP (Sigma-Aldrich, St. Louis, MO) 1 h and *t*BHP 2 h groups according to the length of treatment. The *t*BHP groups were divided into SB203580(+) and SB203580(−) depending on whether or not they were pretreated with SB203580 (Tocris Bioscience, Ellisville, MO) before *t*BHP treatment. Thus, there were five groups: the non-treatment control, the SB203580(−) + *t*BHP 1 h, SB203580(−) + *t*BHP 2 h, SB203580(+) + *t*BHP 1 h, and SB203580(+) + *t*BHP 2 h. The cells were cultured to reach 80% confluence. Then the medium was replaced with DMEM containing 200 μM *t*BHP. The SB203580(+) groups were pretreated with 25 μM SB203580 for 0.5 h before adding *t*BHP. After 1 or 2 h of *t*BHP treatment, the iHTM cell samples were collected for subsequent experiments.

### Cell viability assay

Cells were seeded in 96-well plates (Corning, Cambridge, MA) at a density of 5×10^4^ cells/ml and incubated for 24 h (to 80% confluence). They were then treated with 200 μM *t*BHP, with or without pretreatment with 25 μM SB203580. After culture for another 1 or 2 h, the culture medium was replaced with 200 μl of medium containing 5 mg/ml 3-(4,5-dimethyl-2-thiazolyl) −2,5-diphenyl-2h-tetrazolium bromide (MTT) (Sigma-Aldrich) and incubated for 4 h at 37 °C. Then, all solutions were removed, and the cells and crystallized dyes were dissolved in 150 μl dimethyl sulfoxide (Sigma-Aldrich) per well and shaken on a shaking table bed for 10 min. Absorbance at 570 nm was measured with a micro-plate reader (BioTek Instruments, Winooski, VT).Cell viability was calculated according to the following equation: Cell viability (%)=(OD570_[sample]_/OD570_[control]_) × 100, where OD570_[sample]_ is the average absorbance of the treated cells, and OD570_[control]_ is the average absorbance of the control cells.

### Intracellular ROS level determination

An ROS assay kit (Beyotime, Haimen, China) was used to determine the intracellular ROS levels of iHTM cells. According to the manufacturer’s instructions, at the end of the treatment with SB203580 and *t*BHP, 5×10^6^ cells were collected and resuspended in 10 μM dihydrodichlorofluorescein diacetate (DCFH-DA) with serum-free medium. Intracellular DCFH-DA can be deesterified to dichlorodihydrofluorescein, which is oxidized by ROS to produce the fluorescent compound dichlorofluorescein. After incubation at 37 °C for 30 min, the fluorescence intensity was measured using a flow cytometer (Becton, Dickinson and Company, Franklin Lakes, NJ) at Ex/Em=488/525 nm. (Ex represents the excitation wavelength and Em represents the emission wavelength).

### Proteasome proteolytic activity

Cells were seeded at a density of 5×10^4^ cells/ml in white-walled 96-well plates (Corning) and treated with SB203580 and *t*BHP as described above. To determine the chymotrypsin-like protease activity, Chymotrypsin-like Cell-Based Assays (Promega, Madison, WI) were used according to the manufacturer’s instructions. Briefly, Proteasome-Glo™ Cell-Based Reagents (Proteasome-Glo™ Cell-Based Buffer, Luciferin Detection Reagent), and the succinyl-leucine-leucine-valine-tyrosine- aminoluciferin [Suc-LLVY]-Glo™ Substrate, the sensitive fluorogenic substrate for the 20S proteasome were each prepared and equilibrated at 22 °C for 30 min before use, while the assay plates were simultaneously equilibrated. Ten minutes after the reagent was added, luminescence was determined in terms of relative light units using a multi-plate reader. Each point represents the average of four wells.

### Hoechst 33258 staining for apoptosis detection

Cells were seeded on slides at a density of 5×10^4^/ml in 6-well plates. After treatment as mentioned above, cells of all five groups were washed twice with phosphate-buffered saline (PBS), fixed in 4% paraformaldehyde for 10 min, and then stained with Hoechst 33258 (Invitrogen, Carlsbad, CA) for 5 min. After the cells were washed twice with PBS, they were observed under a fluorescence microscope. The nuclei of living cells were homogeneous blue; those of apoptotic cells were compact, condensed, and whitish blue.

### Annexin V-fluorescein isothiocyanate/propidium iodide double staining assay

Apoptotic cells were quantified using an Annexin V-fluorescein isothiocyanate (FITC)/propidium iodide (PI) kit (BioVision, Palo Alto, CA) and detected by flow cytometry according to the manufacturer’s protocol. Briefly, at the end of treatment with SB203580 and *t*BHP, the cells were detached, collected, resuspended in binding buffer ([pH 7.5] 10 mM HEPES, 2.5 mM CaCl_2_, and 140 mM NaCl), incubated with annexin V-FITC/PI for 10 min in the dark, and then analyzed by flow cytometry. Cells in the early stage of apoptosis stained positive for annexin V-FITC, while those in the late stage of apoptosis stained positive for both annexin V-FITC and PI. The data were analyzed using the Modfit and Cell Quest software programs (Becton, Dickinson and Company).

### Western blot analysis

All cells were washed twice with cold PBS and lysed in 100 μl of protein extraction reagent (Invitrogen). Then, the suspension was centrifuged at 4 °C at 16,000× g for 15 min. Protein concentration was determined using Bio-Rad Protein Assay Kit II (Bio-Rad Lab). The proteins were separated by 10% sodium dodecyl sulfate-PAGE and electrically transferred to polyvinylidene difluoride membranes (Invitrogen). The membranes were blocked with 0.5% skim milk in Tris-buffered saline containing 0.05% Tween-20 for 1.5 h. They were then incubated overnight with anti-p38 and anti-phospho-p38 antibodies (1:1,000 final dilution; Cell Signaling Technology, Boston, MA) and anti-glyceraldehyde-3-phosphate dehydrogenase (GAPDH) antibodies (Sigma-Aldrich) as the loading control. Immunoreactive bands were detected by incubation with a horseradish peroxidase–conjugated secondary antibody and a chemiluminescent substrate (Chemicon, Temecula, CA). The scanned films were densitometrically analyzed using the QuantityOne software (Bio-Rad Lab).

### Statistical analysis

SPSS version 16.0 for Windows (SPSS Science, Chicago, IL) statistical software package was used. All results shown represent means±SD from triplicate experiments performed in a parallel manner. Statistical significances were evaluated using the Student’s *t*-test and one-way ANOVA. Results were considered significant at the p<0.05 level.

## Results

### Morphological changes in tBHP-treated cells

**Figure 1:**
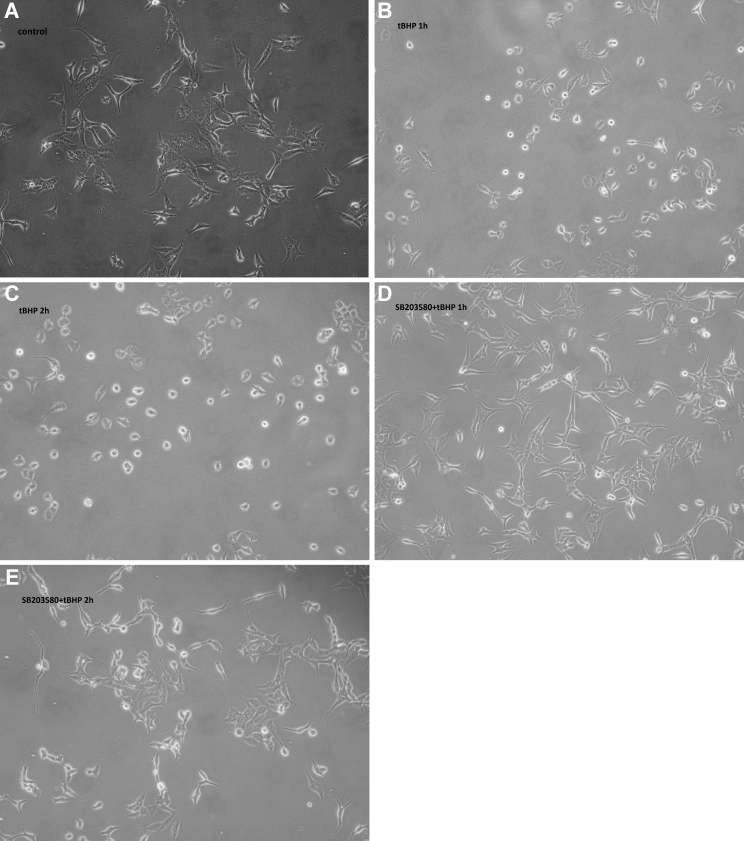
Morphology of iHTM cells after *t*BHP exposure for 1 h and 2 h with or without pretreatment with SB203580. iHTM cells were not treated with *t*BHP or SB203580 (**A**). Cells were not pretreated with SB203580 and just treated with *t*BHP for 1 h (**B**) and 2 h (**C**). Cells were pretreated with SB203580 for 30 min and then treated with *t*BHP for 1 h (**D**) and 2 h (**E**). More cells from SB203580(-) group became rounded, shrank and dettached after treatment with 200 μM *t*BHP for 1 h as compared to those from SB203580(+) group. Gross morphological changes of cells were more apparent after *t*BHP treatment for 2 h. Phase-contrast light microscope 100×.

The iHTM cells were cultured and treated as described above. After *t*BHP treatment, morphological changes in the cells were assessed using phase-contrast light microscopy. As shown in Figure 1A, iHTM cells in control group were bright, fusiform and firmly adherent. After incubation with *t*BHP for 1 h, most SB203580(-) cells shrank, turned round, and became loosely attached (Figure 1B). However, the changes in cell morphology were milder in the SB203580(+) group (Figure 1D). Further, the differences in morphology were more apparent after prolonged *t*BHP treatment (Figure 1C,E).

### Changes in cell activity

**Figure 2:**
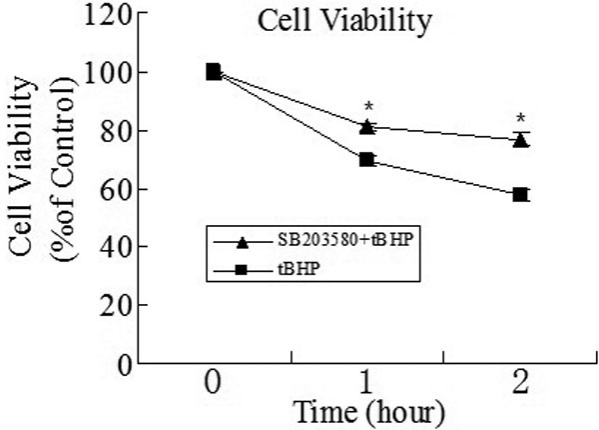
Detection of cell viability by MTT assay after *t*BHP exposure. After exposure to *t*BHP for 1 h, the cell viability of the SB203580(-) group was 69.35%±1.52%, while that of the SB203580(+) group was 81.08%±1.93%. After 2 h, the cell viabilities were 57.88%±2.20% and 76.72%±2.11% in the SB203580(-) and SB203580(+) group, respectively. The viability of SB203580(+) cells were significantly higher than that of SB203580(-) cells after exposure to *t*BHP. The data were presented as mean±SD, n=4, p<0.05. Data were presented as the percentage of viable cells compared with untreated (control) cells.

Cell viability was tested using MTT assay. As shown in Figure 2, after incubation with *t*BHP for 1 h, the viability of the SB203580(−) and (+) cells decreased by 30.70%±1.5% and 18.92%±1.9%, respectively. After *t*BHP treatment for 2 h, the viabilities of SB203580(−) and (+) cells were reduced by 42.12%±2.2% and 23.28%±2.1%, respectively. Both groups showed significant differences between SB203580(−) and (+) cells (p=0.001 in 1 h group, p=0.000 in 2 h group).

### Assay of intracellular ROS level

**Figure 3:**
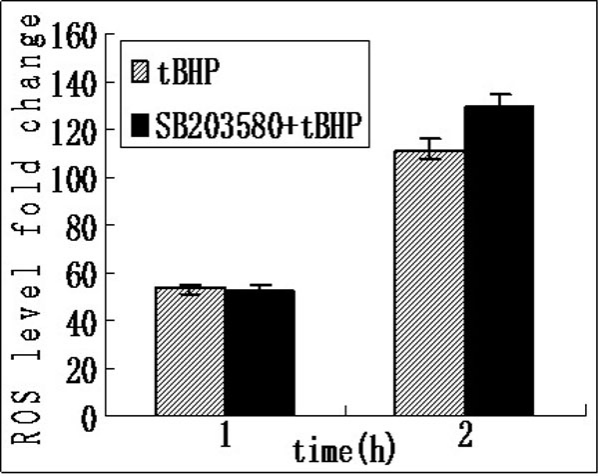
ROS levels in iHTM cells after exposure to *t*BHP with or without pretreatment with SB203580. The ROS levels of the SB203580(-) cells and SB203580(+) cells increased 53.47±1.75 and 52.64±2.32 folds after *t*BHP exposure for 1 h. Those of the SB203580(-) cells and SB203580(+) cells increased 111.81±4.33 and 130.48±4.21 folds after *t*BHP exposure for 2 h. The data were presented as mean±SD, n=4, p>0.05. Data were expressed as a fold change in ROS production of the cells. ROS level in iHTM cells without *t*BHP and SB203580 was set as 1.

Fluorescent analysis with DCFH-DA was used to investigate the intracellular ROS in iHTM cells. The fluorescence intensity was measured by flow cytometry. As shown in Figure 3, *t*BHP significantly increased the ROS level in both SB203580(−) and (+) cells. Compared to the control group, after *t*BHP exposure for 1 h, the ROS level of SB203580(−) and SB203580(+) cells increased by 53.47±1.75 and 52.64±2.32 folds; after *t*BHP exposure for 2 h, the ROS level of SB203580(−) and SB203580(+) cells increased by 111.81±4.33 and 130.48±4.33 folds, respectively. The difference in the ROS levels between the SB203580(−) and SB203580(+) cells showed no statistical significance (p=0.499 in 1 h group, p=0.071 in 2 h group).

### Measurement of chymotrypsin-like proteasome activity

**Figure 4:**
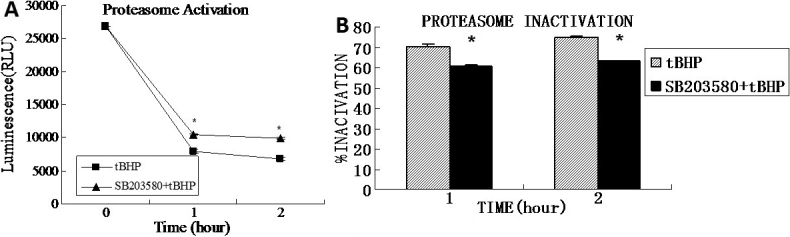
Chymotrypsin-like preoteasome activity in iHTM cells after exposure to *t*BHP with or without pretreatment with SB203580. The luminescence intensity of the control cells was 26869±1156.353. After *t*BHP exposure for 1 h, the luminescence intensities of the SB203580(−) and SB203580(+) cells were 7901.95±236.98 and 10493.25 ±146.49; and those of the SB203580(−) and SB203580(+) cells were 6734.9±273.23 and 9943 ±111.23 (**A**). After *t*BHP exposure for 1h, the chymotrypsin-like proteasome inactivations in SB203580(-) cells and SB203580(+) cells were 70.59±0.88 and 60.94±0.55. After *t*BHP exposure for 2 h, those in SB203580(-) cells and SB203580(+) cells were 74.93±0.54 and 62.99±0.41 respectively (**B**). The data were presented as mean±SD, n=4, p<0.05.

To determine the changes of the levels of proteasome activity in iHTM cells after SB203580 and *t*BHP treatment, the chymotrypsin-like proteasome activity of the 20S proteasome was measured. The results are shown in Figure 4. The luminescence intensity of the control cells was 26869±1156.353. After *t*BHP exposure for 1 h, the luminescence intensities of the SB203580(−) and SB203580(+) cells were 7901.95±236.98 and 10493.25 ±146.49; and those of the SB203580(−) and SB203580(+) cells were 6734.9±273.23 and 9943 ±111.23 after *t*BHP exposure for 2 h (Figure 4A). Compared to the control group, after *t*BHP exposure for 1 h, the chymotrypsin-like proteasome inactivations were 70.59%±0.88% in the SB203580(−) cells, and 60.94%±0.55% in the SB203580(+) cells; after *t*BHP exposure for 2 h, those in the SB203580(−) cells and SB203580(+) cells were 74.93%±0.54% and 62.99%±0.41%, respectively (Figure 4B). The activity of the 20S proteasome reduced after *t*BHP treatment, although this decrease was slightly lower in cells pretreated with SB203580 (p=0.000 in both of the 1 h and 2 h groups).

### Effects of tBHP and SB203580 on apoptosis and cell death

**Figure 5:**
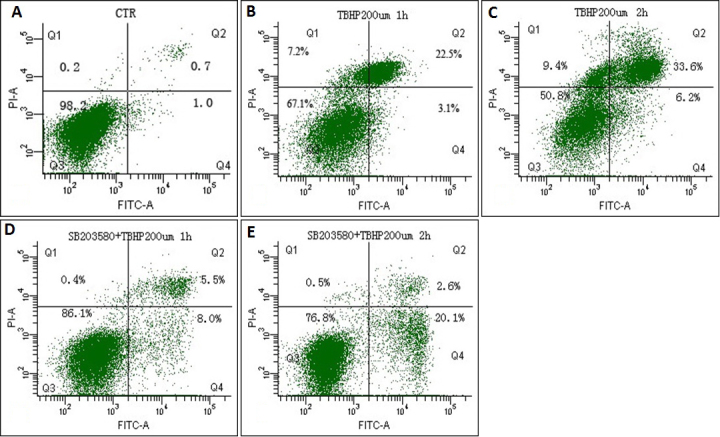
Apoptosis of iHTM cells detected by flowcytometry after *t*BHP exposure with or without pretreatment with SB203580. iHTM cells not treated with *t*BHP or SB203580 were set as control (**A**). Cells treated with tBHP for 1 h (**B**) and 2 h (**C**) without SB203580 pretreatment. Cells were pretreated with SB203580 for 30 min and then treated with tBHP for 1 h (**D**) and 2 h (**E**). The rate of apoptosis in SB203580(-) cells and SB203580(+) cells after exposure to *t*BHP for 1 h were 28.23±3.23% (**B**) and 12.75±1.91% (**D**), those in SB203580(-) cells and SB203580(+) cells after exposure to *t*BHP for 2 h were 39.20±5.91% (**C**) and 20.40±3.44% (**E**). The rate of cell death and apoptosis in SB203580(-) cells were higher than SB203580(+) cells (p<0.05). The data were presented as mean±SD, n=4, p<0.05.

**Figure 6:**
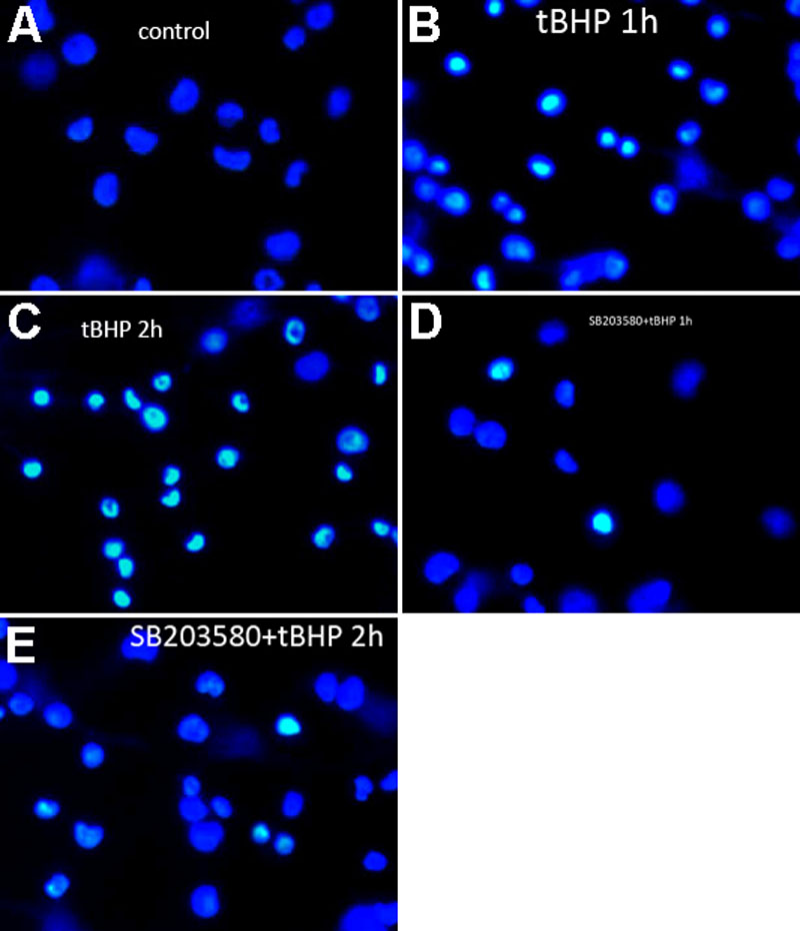
The iHTM cell nuclei staining by Hoechst33258 for apoptosis detection after *t*BHP exposure with or without pretreatment with SB203580. iHTM cells not treated with *t*BHP or SB203580 were set as control (**A**). The nuclei of control cells were homogeneously blue after staining by Hoechst33258 (**A**). After exposure of tBHP for 1 h without pretreatment with SB203580, many cells were apoptotic. The nuclei of apoptotic cells were compact and condensed, which were heterogeneously stained (**B**). After 2 h, more cells were apoptotic (**C**). However, in the cells pretreated with SB203580, the percentages of apoptotic cells were less (**D** and **E**).

We investigated apoptosis and cell death in all cell groups using flow cytometry and confocal microscopic analysis. The results are shown in Figure 5 and Figure 6. Compared to the control group, after *t*BHP exposure for 1 h, the apoptosis in the SB203580(−) cells and the SB203580(+) cells were 28.23%±3.23% and 12.75%±1.91% separately; after *t*BHP exposure for 2 h, the apoptosis in the SB203580(−) cells and the SB203580(+) cells were 39.20%±5.91% and 20.40%±3.44% separately. Obviously, cells pretreated with SB203580 showed a significantly lower extent of cell death and apoptosis after *t*BHP treatment than those not pretreated with SB203580 (p=0.002 in 1 h group, p=0.009 in 2 h group).

### Assay of p38MAPK and phospho-p38MAPK levels

**Figure 7:**
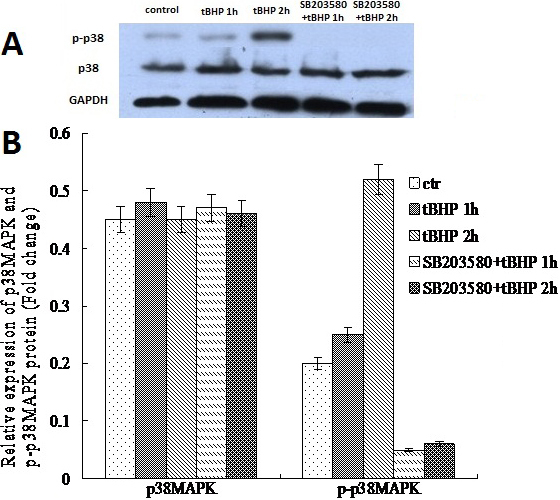
Western blot analysis of p38 MAPK in *t*BHP treated iHTM cells. The p38MAPK and phosphorylated p38MAPK were detected by western blotting with p38 MAPK (p38) and phospho-p38 MAPK (p-p38) antibodies. The same samples were probed with GAPDH as a loading control (**A**). The p38MAPK levels were of little difference among all cells. The p-p38 levels increased after exposure to *t*BHP without pretreatment with SB203580, especially in the cells treated for 2 h. Nevertheless, in the SB203580 (+) groups, the level of p-p38 substantially decreased. There were nearly no p-p38 could be detected in the cells pretreated with SB203580 (**B**).

To assess whether the p38MAPK pathway was activated in iHTM cell apoptosis induced by *t*BHP, we measured the amounts of the p38MAPK and phospho-p38MAPK proteins using western blot. The p38MAPK and phospho-p38MAPK proteins in control cells were 0.455±0.045 and 0.21±0.014. After *t*BHP exposure for 1 h, the p38MAPK in the SB203580(−) cells and the SB203580(+) cells were 0.482±0.031 and 0.475±0.044 separately; after *t*BHP exposure for 2 h, this protein in the SB203580(−) cells and the SB203580(+) cells were 0.456±0.023 and 0.461±0.021 separately. No differences were found among the control, SB203580(−), and SB203580(+) cells in terms of the total p38MAPK level before and after *t*BHP exposure (p>0.05). Yet the activated form of p38MAPK, i.e., phospho-p38MAPK, increased significantly after *t*BHP exposure in SB203580(−) cells than those in control and SB203580(+) cells. After *t*BHP exposure for 1 h, the phospho-p38MAPK in the SB203580(−) cells and the SB203580(+) cells were 0.25±0.03 and 0.05±0.026 separately (p=0.001); after *t*BHP exposure for 2 h, the phospho-p38MAPK protein were 0.51±0.023 and 0.06±0.014 separately (p=0.000). Compared to the control cells, the phospho-p38MAPK protein expression were decreased in SB203580(+) cells (p=0.001 for 1 h and 2 h *t*BHP exposure; Figure 7).

## Discussion

The reasons for the increase in the outflow resistance of the TM–SC conventional outflow tissue in POAG patients remain unclear. Many studies have shown that oxidative stress plays elementary roles in the pathogenesis of POAG [4-6,20]. ROS are reported to trigger degeneration in human TM and its endothelial cell components. p38MAPK signaling pathway might play a role in the adverse effect of ROS, since kinase inhibitors increased cell viability in the current study, which was consistent with the previous studies in intestinal epithelial cell and HL-60 cells [21,22].

Molecular oxygen (O_2_) can be reduced to form the anion superoxide radical (O_2_^-^), and subsequently hydrogen peroxide (H_2_O_2_) and hydroxyl radical (OH^-^). The superoxide anion attacks proteins, DNA, and other macromolecules, and subsequently leads to degeneration of TM. Previous studies have shown that H_2_O_2_ can promote apoptosis of TM cells [8]. However, the oxidative effect of H_2_O_2_ is short and unstable. Compared with H_2_O_2_, the oxidative effect of *t*BHP lasts longer. Thus, we chose *t*BHP as the oxidative inducer. In the current study, we found that the intracellular ROS levels of iHTM cells increased significantly after exposure to *t*BHP in the presence and absence of MAP kinase inhibitor, which implied that the *t*BHP induced oxidative stress model in iHTM cells is valid. And the apoptosis of iHTM cells after *t*BHP incubation increased consistently with ROS levels, which confirmed the ROS toxicity to iHTM cells.

Recently, the role of p38MAPK signaling pathway in oxidative stress has drawn the attentions of researchers [14,15]. Several studies indicated that the activation of p38MAPK plays an important role in apoptosis induced by various stimuli. For example, p38MAPK activation is involved in apoptosis induced by nerve growth factor stimulation in PC12 neuronal cells, while SB203580 pretreatment was shown to inhibit apoptosis in primary neurons [23]. The inhibition of p38MAPK by ebselen protects neuronal cells from NO cytotoxicity [24]. In the current study, we found that the level of phospho-p38MAPK, an active mode of p38MAPK, increased in iHTM cells after *t*BHP treatment. Pretreatment of the cells with SB203580, an inhibitor highly specific for p38MAPK both in vitro and in vivo, functionally inhibited phospho-p38MAPK. Further, *t*BHP-induced apoptosis in iHTM cells was effectively prevented by SB203580 pretreatment. These findings suggest that the activation of the p38MAPK signaling pathway mediates *t*BHP-induced apoptosis.

In the current study, ROS levels increased significantly after *t*BHP treatment, and pretreatment with p38MAPK inhibitor SB203580 had little influence on the ROS levels, although it inhibited p38MAPK phosphorylation and alleviated cell injury. These findings suggest that *t*BHP-induced ROS release occurs upstream of p38MAPK, which is consistent with previous reports showing that p38MAPK is responsive to ROS and involved in cell apoptosis. For example, ROS may be responsible for p38MAPK activation induced by tissue growth factor-beta [25], and H_2_O_2_ preferentially stimulates the phosphorylation of p38MAPK in vascular smooth-muscle cells [26].

We also investigated the chymotrypsin-like proteasome activity of the 20S proteasome, which was reported to protect eukaryotic cells from oxidative stress by eliminating the misfolded proteins generated by direct ROS damage [27]. In the current study, we found that the chymotrypsin-like proteasome activity declined sharply after *t*BHP treatment. Two mechanisms might lead to the impairment of the proteasome by oxidative stress as Caballero [28] mentioned: saturation of the proteasome by an excessive number of misfolded proteins, and direct oxidation of proteasome components. On the other hand, with SB203580 pretreatment, the decreased proteasome activity was slightly reversed, which implied that the proteins downstream to phospho-p38MAPK could not be activated after blockage of the p38MAPK phosphorylation by SB203580, and then the number of misfolded proteins were minimized.

There were some limitations in the current study. While the survived TM cells showed normal morphology, whether their functions remained normal was still unclear. Further study on the effect of blockage of the p38MAPK signaling pathway on the functions of TM cells is necessary. Moreover, the cell viabilities 1 and 2 h after *t*BHP treatment with or without SB203580 pretreatment were explored in the current study. However, apoptosis of TM cells might reach its peak several hours after the injury. Further study on the longer effects of *t*BHP and SB203580 on the TM cells should be performed.

In summary, our study shows that *t*BHP-induced apoptosis is related to an ROS-activated p38MAPK signaling pathway in TM cells. Blockage of p38MAPK phosphorylation can reduce cell injury and apoptosis significantly. Thus, further study of the mechanisms of p38MAPK signaling pathway in TM cell apoptosis may shed light on its pathogenesis for POAG.
